# Complicated pulmonary human coronavirus-NL63 infection after a second allogeneic hematopoietic stem cell transplantation for acute B-lymphocytic leukemia: A case report

**DOI:** 10.1097/MD.0000000000026446

**Published:** 2021-06-25

**Authors:** Zhihui Li, Shuo Meng, Qinlong Zheng, Tong Wu

**Affiliations:** a Department of Bone Marrow Transplantation; b Laboratory of Molecular Diagnostics, Beijing Boren Hospital, Beijing, China.

**Keywords:** acute B-lymphocytic leukemia, complications, high-throughput sequencing of infectious pathogen macrogenome, human coronavirus NL63, post-second allogeneic hematopoietic stem cell transplantation

## Abstract

**Rationale:**

Viruses are the most common pathogens that can cause infection-related non-recurrent death after transplantation, occurring mostly from the early stages of hematopoietic stem cell transplantation (HSCT) to within 1 year after transplantation. Human coronavirus (HCoV)-NL63 is a coronavirus that could cause mortality among patients with underlying disease complications. Serological tests are of limited diagnostic value in immunocompromised hosts and cases of latent infection reactivation. In contrast, macro-genomic high-throughput (DNA and RNA) sequencing allows for rapid and accurate diagnosis of infecting pathogens for targeted treatment.

**Patient concerns:**

In this report, we describe a patient who exhibited acute B-lymphocytic leukemia and developed complicated pulmonary HCoV-NL63 infection after a second allogeneic HSCT (allo-HSCT). Six months after the second allo-HSCT, he developed sudden-onset hyperthermia and cough with decreased oxygen saturation. Chest computed tomography (CT) suggested bilateral multiple rounded ground-glass opacities with the pulmonary lobules as units.

**Diagnoses:**

HCoV-NL63 was detected by metagenomic next-generation sequencing (NGS), and HCoV-NL63 viral pneumonia was diagnosed.

**Interventions:**

The treatment was mainly based on the use of antiviral therapy, hormone administration, and gamma-globulin.

**Outcomes:**

After the therapy, the body temperature returned to normal, the chest CT findings had improved on review, and the viral copy number eventually became negative.

**Lessons:**

The latest NGS is an effective method for early infection diagnosis. The HCoV-NL63 virus can cause inflammatory factor storm and alter the neutrophil-to-lymphocyte ratio (NLR). This case suggests that the patient's NLR and cytokine levels could be monitored during the clinical treatment to assess the disease and its treatment outcome in a timely manner.

## 1 Introduction

Coronaviruses are RNA viruses comprising 7 species that are currently known to infect humans, all of which are alpha (HCoV-229E and HCoV-NL63) and beta (HCoV-OC43, HCoV-HKU1, SARS-CoV, MERS-CoV, and SARS-CoV-2) types. In 2004, human coronavirus (HCoV)-NL63 was isolated from a patient with upper respiratory tract infection.^[1]^ HCoV-NL63 infection is usually community-acquired and mostly transmitted through airborne droplets. It usually causes symptoms of mild upper respiratory tract infections,^[2]^ with fever, cough, rhinitis, and sore throat as the main clinical symptoms. The clinical manifestations mainly include upper respiratory tract infections, bronchitis, bronchopneumonia, bronchiolitis, and pseudomodel laryngitis.^[3]^ HCoV-NL63 infection is a self-limiting disease, in which the functional immune response can completely eliminate the virus in healthy individuals. Our literature summary of over the last 10 years showed that the HCoV-NL63 detection rates in immunocompetent populations were 0.1% to 16.5%.^[4–22]^ Other studies have shown mortality following the HCoV-NL63 infection but almost always related to underlying disease complications, such as malignancy, renal failure, immunosuppression, and diabetes mellitus.^[23–25]^ The occurrence of HCoV-NL63 infection is not sex-, age-, or geography-specific and accounts for approximately 5% of all acute respiratory disease cases. These data show that HCoV-NL63 infection cannot be ignored.

## 2 Case report

**Figure 1:**
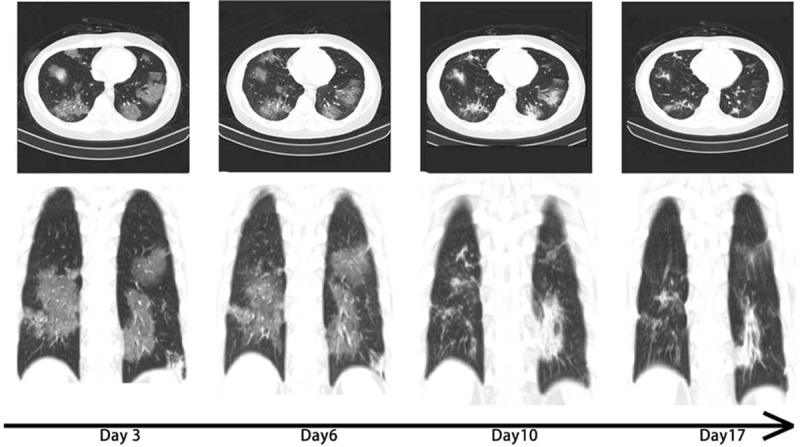
Plain chest CT scan of the patient. Fever was registered on the day of admission, with multiple block-like and patchy shadow interstitial changes in both lungs on Day 3. A gradual absorption of the pulmonary lesions could be detected during the course of the treatment. Therefore, the treatment was considered effective, and the condition had improved. CT = computed tomography.

**Figure 2:**
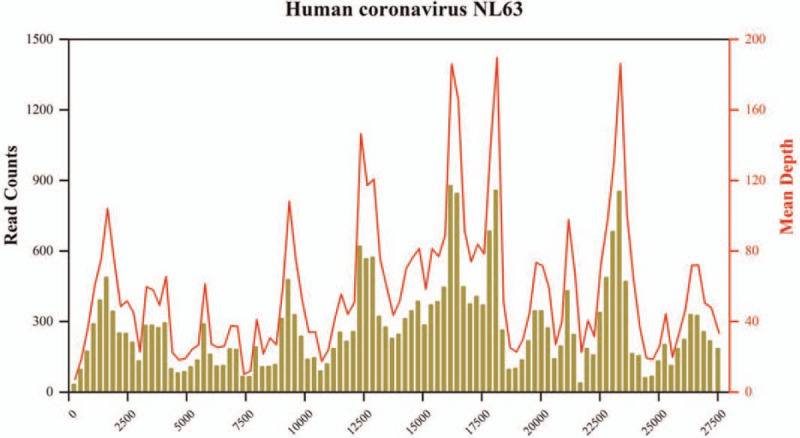
mNGS analysis detected HCoV-NL63: non-human sequences were compared with the HCoV-NL63 reference genome with a unique sequence count of 38,636, coverage of 96.4%, and an average depth of 75.8X. HCoV = human coronavirus, mNGS = metagenomic next-generation sequencing.

**Figure 3:**
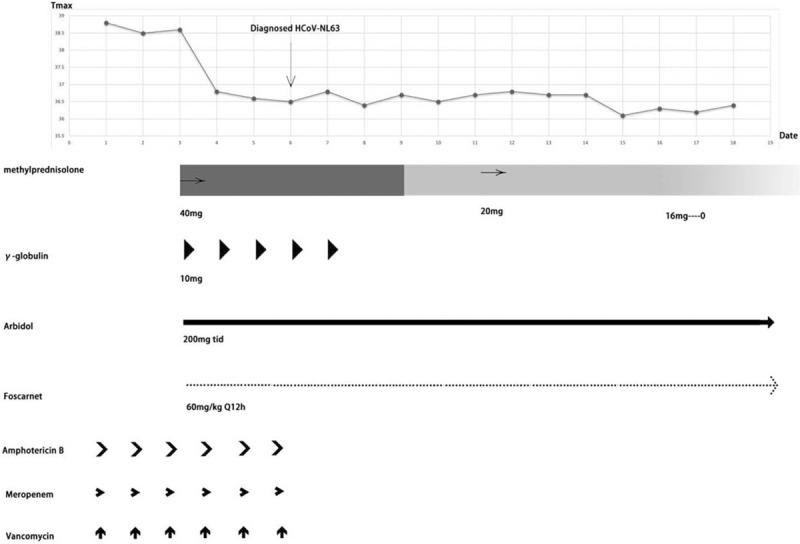
The first day of the patient with fever was defined as Day 1. The patient was treated with broad-spectrum antibiotics, specifically meropenem, vancomycin, and amphotericin B. The treatment effect was suboptimal, and the patient remained persistently hyperthermic without significant fever peak change from the previous measurement. Antiviral therapy with methylprednisolone, gamma globulin, foscarnet sodium, and arbidol was administered from Day 3. The temperature decreased to normal from Day 4, and no further fever could be observed during the subsequent treatment. On Day 6, the diagnosis of HCoV-NL63 virus pneumonia was considered definitive based on the NGS results, and treatment with meropenem, vancomycin, and amphotericin B was discontinued. Glucocorticoid use was routinely reduced until discontinuation. The patient was discharged on Day 18 with improvement and foscarnet sodium discontinuation. Oral Arbidol was continued after discharge and discontinued after 5 days. HCoV = human coronavirus, NGS = next-generation sequencing.

A 20-year-old man was diagnosed with acute B-lymphocytic leukemia in June 2016. The bone marrow morphology studies indicated that the disease had been in remission after the induction therapy provided on July 5, 2016, while the flow cytometry results remained positive. Allogeneic hematopoietic stem cell transplantation (allo-HSCT) was performed on November 3, 2016, with his elder sister as the fully human leukocyte antigen (HLA) 10/10- and blood type A-matched sibling donor. Leukocytes and platelets were successfully implanted at days +14. The implantation survival identification at days +28 was as follows: morphology in remission, negative flow cytometry results, complete donor type in the bone marrow, and peripheral blood chimerism. The re-examination of the bone marrow morphology in November 2017 showed the presence of 15% naïve lymphocytes, suggesting hematological relapse. Positron emission tomography/computed tomography (PET/CT) showed extramedullary relapse. After fluorine pyrimidine and carboplatin regimen chemotherapy, the re-examination showed bone marrow remission, without abnormalities in the cerebrospinal fluid. On January 3, 2018, 1 × 10^5^/kg of CD19-chimeric antigen receptor T-cell immunotherapy cells were re-infused into the patient, and the re-examination showed bone marrow remission. The bone marrow and cerebrospinal fluid of the patient continued to be in remission after re-infusion of 2 × 10^5^/kg of CD22-chimeric antigen receptor T-cell immunotherapy cells on July 10, 2018. The patient presented a small nodule on the left foot sole without pain or local erythema, in December 2019. Pathological biopsy of the plantar mass revealed a small round cell malignancy, considered to be leukemic cell infiltration. Flow cytometry showed that 88.43% of the analyzable CD19+/CD45- cells were naïve B lymphocytes with abnormal phenotypes, expressing CD34, CD10bright, CD22, CD81dim, CD58, and HLA-DR. In January 2020, PET–CT re-examination suggested multiple extramedullary leukemic infiltrates, and CD22 monoclonal antibody was successively administered twice. On May 6, 2020, PET–CT of the lower extremities showed incomplete remission of extramedullary lesions. A second allo-HSCT was initiated on May 9, 2020, with a haploidentical HLA 6/10-matched maternal donor and donor and recipient blood types O and A, respectively. The pretreatment protocol was cytarabine/fludarabine/thiotepa/anti-thymus immunoglobulin/*N*-(2-chloroethyl)-*N*′-(4-methylcyclohexyl)-*N*-nitrosourea, and the procedure went well. Leukocytes and platelets were viable at days +11 and +16, respectively. The re-examination by bone marrow puncture biopsy 1 month after the transplantation revealed that the primary disease was relieved, and bone marrow and peripheral blood were of full donor chimera. The re-examination 2 months after the transplantation showed complete remission of the bone marrow and cerebrospinal fluid. PET–CT showed that the extensive multiple hypermetabolic foci in both lower extremities and feet had partially reduced compared with those in the previous examination. Considering that the leukemic infiltrate-related results had partially improved and the residual tumor metabolic activity was still significant, local radiotherapy was provided to the lower legs and soles of the feet. In mid-November 2020 (+6 months after transplantation), a follow-up visit at another hospital showed hyperthermia without any obvious cause, with cough but without phlegm. The maximum temperature was 39.8 °C, and the minimum blood oxygen saturation was 85%. No positive signs were found on the chest during physical examination. The results of the routine blood tests were as follows: white blood cell count, 7.8 × 10^9^/L; neutrophil count, 5.68 × 10^9^/L; lymphocyte count, 1.06 × 10^9^/L; C-reactive protein, 268.69 mg/L (reference range: 0–10); and procalcitonin, 0.35 ng/mL (reference range: 0–0.07). The results of the G-test, GM-test, and TSPOT, as well as those of the tests for *Mycoplasma pneumoniae* antibody Immunoglobulin M (IgM), *Chlamydia pneumoniae* antibody IgM, influenza A virus antibody IgM, influenza B virus antibody IgM, parainfluenza virus antibody IgM, respiratory syncytial virus and influenza virus antibody IgM, adenovirus antibody IgM, coxsackie B virus antibody IgM, *Legionella pneumophila* antibody IgM, novel coronavirus antibody IgM, novel coronavirus antibody IgG, peripheral blood Epstein-Barr virus, cytomegalovirus virus, and novel coronavirus nucleic acid copy number, were negative. A mass shadow could be detected on the chest CT (Day 3 in Fig. 1). No positive pathogens were detected by metagenomic next-generation sequencing (mNGS, DNA+RNA) in the peripheral blood specimens. The pharyngeal swab mNGS detected 10 *Streptococcus pneumoniae*-specific sequences, 2 *Enterococcus faecalis*-specific sequences, and 3 *Aspergillus flavus*-specific sequences and common upper respiratory flora in the DNA library; in the RNA library, 28,636 HCoV-NL63-specific sequences (Fig. 2) and common respiratory flora were detected. The coverage of HCoV-NL63 was 96.4%, the average depth was 75.8X, and the relative abundance was 99.4%, which satisfied the quality control requirements, and the test results were in full agreement with the clinical features. The final diagnosis was established as HCoV-NL63 viral pneumonia. Intravenous infusion of foscarnet sodium 60 mg/kg q12 h and methylprednisolone 40 mg qd, as well as oral antiviral therapy with Arbidol 200 mg tid, were administered, with gradual reduction after 7 days. Furthermore, gamma globulin 0.4 g/kg was administered for 5 days as an immunoregulatory therapy (Fig. 3). As a result, the symptoms of the patient improved significantly: the body temperature decreased to normal, no fever peak appeared, and blood oxygen was stably maintained at 97% to 99% without nasal cannula oxygenation. The chest CT showed significant and slightly more resorbed lesions than before (Fig. 1). Multiple re-examinations of cytokines during the onset and treatment period showed that as the body temperature decreased, the cough symptoms improved. The patient showed improvement and was discharged.

## 3 Discussion

In allo-HSCT, infections can cause nonrecurrent death after transplantation, occurring mostly from the early stages of HSCT to within 1 year after transplantation. Viruses are the most common pathogens. Among them, cytomegalovirus is the most common source of viral infection after transplantation,^[26]^ and the others include adenoviruses, bocaviruses, coronaviruses, human herpesvirus-6, and the West Nile virus.^[27]^ Serological tests are of limited diagnostic value in immunocompromised hosts and cases of latent infection reactivation. The mNGS (DNA and RNA) method, that is, macro-genomic high-throughput sequencing of infecting pathogenic microorganisms,^[28]^ allows for the rapid and accurate diagnosis of infecting pathogens for targeted treatment. The mNGS approach allows the testing of various specimens, such as blood, pharyngeal swabs, and alveolar lavage fluid. By extracting total DNA and RNA from the specimens, we conducted pretreatment and NGS library construction, respectively, followed by double-end 150 bp and single-end 75 bp sequencing using the Illumina platform Nextseq550 sequencer, quality control of the logout data, dehumanized sequence processing, and comparison with the self-constructed pathogenic microorganism reference genome database for further analysis to determine infection-related pathogenic microorganisms. No pathogenic DNA or RNA positive results consistent with the clinical symptoms were found in the peripheral blood mNGS test results of the patient. However, the pharyngeal swab test revealed that the number of HCoV-NL63-specific sequences in the RNA library was 28,636, and the HCoV-NL63 coverage and sequencing depth met the quality control requirements (Fig. 2). The test results were fully consistent with the clinical conditions. The diagnosis was established as HCoV-NL63 viral pneumonia.

This viral infection is self-limiting, with currently no definitively effective antiviral treatment. After the administration of hormones, gamma globulin, and antiviral therapy, the body temperature returned to normal. Furthermore, symptoms such as cough and expectoration improved, and the lung imaging results suggested the gradual absorption of the interstitial changes. Pharyngeal swab samples were re-examined using NGS on day 5 of the treatment, revealing that the number of HCoV-NL63-specific sequences was reduced to 284, and the level of cytokines in the body was significantly reduced. This result indicates that the coronavirus infection-induced inflammatory factor storm was under control. Mayer et al^37^ reported the case of a 27-year-old woman diagnosed with T-cell acute lymphoblastic leukemia who had a severe complicated pulmonary infection in the agranulocytosis phase of chemotherapy and whose disease was not controlled by antifungal therapy.^[29]^ Analysis of alveolar lavage fluid sample led to the detection of HCoV-NL63, after which interferon was administered, and the viral copy numbers became negative. However, she eventually died due to diffuse alveolar hemorrhage. In a multicenter clinical trial of pathogen detection in 434 post-allo-HSCT patients with CoV infection,^[30]^ the detection rate of NL63 by multiplex Polymerase chain reaction was 16.5%. The rapid and highly sensitive screening diagnosis of emerging viral infections and timely symptomatic treatment can be effective in improving patient survival.

**Figure 4:**
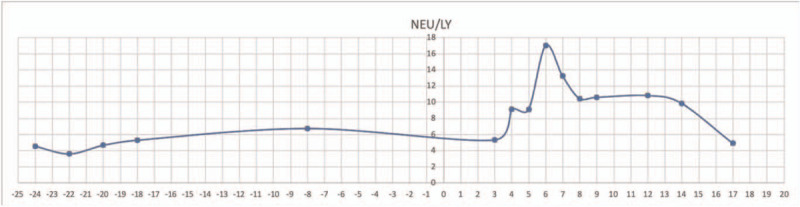
The patient showed a stable peripheral blood neutrophil-to-lymphocyte ratio measured within 1 month prior to admission, with a trend of increasing NEU/LY ratio after the onset of fever (Day 1), peaking on Day 6, decreasing from Day 7 onward, and returning to the level before the disease onset on Day 17.

In the present case, the patient was 6 months post-second-allo-HSCT and had regular blood monitoring before disease onset, with stable peripheral blood neutrophil-to-lymphocyte ratio (NLR). The NLR is an inflammatory marker reflecting systemic inflammation. After the fever onset (day 1), the NLR tended to increase, peaking on day 6, decreasing from day 7 onward, and returning to the level before disease onset on day 17 (Fig. 4). Severe viral infections lead to endothelial cell destruction and production of large amounts of inflammatory factors, such as Interleukin 6, Interleukin 8, Tumor Necrosis Factor-α, and granulocyte colony-stimulating factors, which can stimulate the production of high levels of neutrophils.^[31]^ The inflammation stimulates neutrophil production and accelerates lymphocyte apoptosis. Excessive neutrophil aggregation and activation, as well as the release of toxic enzymes, aggravate alveolar epithelial damage, leading to severe pulmonary edema, hypoxemia, and the eventual development of multiple organ dysfunction syndrome and acute respiratory distress syndrome. NLR is a practical and valid marker for disease progression and prognosis assessment in studies on coronavirus disease 2019.^[32]^ In this case, NLR changes were consistent with the disease course of the patient and his recovery after the treatment. Thus, NLR monitoring can be considered as a rapid way to evaluate treatment efficacy for this viral pneumonia. A certain lag is present in the NLR changes compared with those of cytokines.

**Figure 5:**
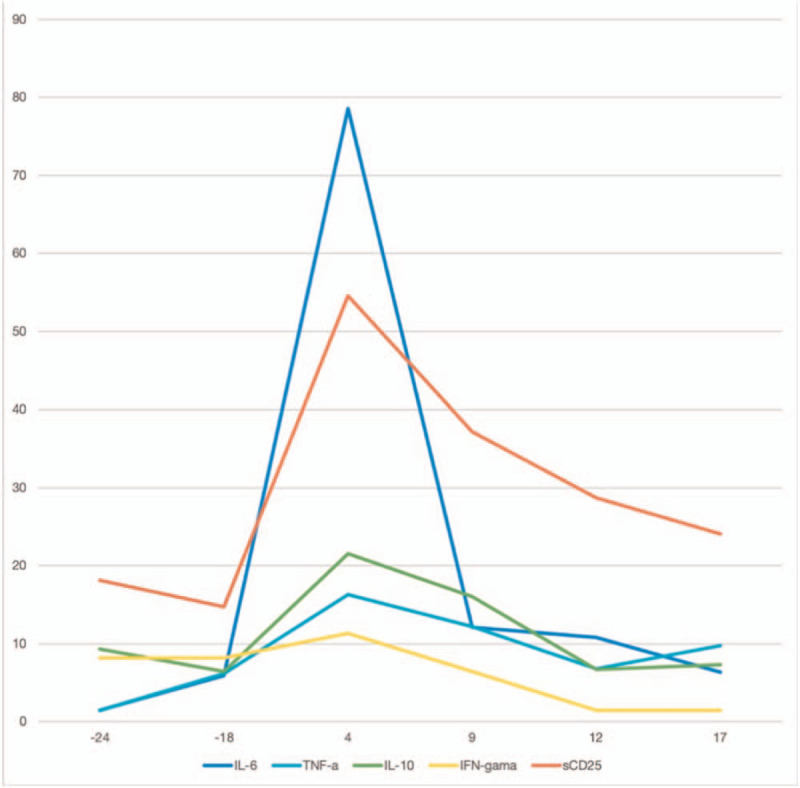
Trends in cytokine levels over the course of the disease, with Interleukin 6, Tumor Necrosis Factor-a, Interleukin 10, and IFN-gamma units in pg/mL and sCD25 unit in 100 pg/mL.

The major high fatality risks, whether in SARS-CoV-2, SARS-CoV, or HCoV-NL63, as reported in this case, arise from multiorgan injury/failure due to the severe cytokine storm associated with viral infections.^[33]^ Cytokine-induced tissue injury or acute phase physiological changes and/or immune cell-mediated responses can rapidly progress to disseminated intravascular coagulation complicated by vascular obstruction or hemorrhage, respiratory distress, hypoxemia, acute respiratory distress syndrome, hypotension, vasodilatory shock, and death. Severe cytokine storms can also lead to renal failure, acute liver injury or cholestasis, and cardiomyopathy.^[34]^ Interferon-γ, interleukin-1, interleukin-6, TNF, and interleukin-18 are key cytokines often considered to have an immunological role in cytokine storms. The changes in the cytokine levels in our patient (Fig. 5) could be observed in the case of Interleukin 6, Tumor Necrosis Factor-a, Interleukin 10, IFN-gamma, and sCD25 from the first day of fever with varying degrees of elevation, peaking on day 4, and subsequently decreasing to normal levels. This suggests that cytokine monitoring is critical in managing virus-associated pneumonia. The progression and treatment outcome of the disease can be assessed based on cytokine levels.

## 4 Conclusion

Patients with malignant hematologic neoplasms are immunocompromised after transplantation, particularly those with a second transplant who have undergone local radiation therapy and are prone to serious infections. Serological examinations might be of limited value in the diagnosis of immune-compromised hosts and cases of latent infection reactivation. In this study, the latest NGS methods were used to extract total DNA and RNA from the specimen for early diagnosis. During the treatment of the patient in this case study, as the inflammatory factor storm was controlled, neutrophil and lymphocyte levels gradually returned to normal, cytokine levels decreased, the disease was controlled, and the viral copy number eventually became negative. This suggests that the NLR and cytokine levels of the patient can be monitored during clinical treatment to assess the disease and its treatment outcome in a timely manner.

## Acknowledgments

The authors thank Professor Li Hongjun, MD, PhD, director of the medical imaging center of Beijing You’an Hospital Affiliated to Capital Medical University, for the analysis and judgment of the imaging results of the patient.

They thank Professor Zhang Bo, MD, PhD, director of the respiratory and infection diagnosis and treatment consultation center of the Gaobo medical group, for the diagnosis and treatment.

## Author contributions

**Conceptualization:** Tong Wu.

**Data curation:** Zhihui Li, Shuo Meng, Qinlong Zheng.

**Investigation:** Shuo Meng, Qinlong Zheng.

**Project administration:** Tong Wu.

**Writing – original draft:** Zhihui Li, Shuo Meng.

**Writing – review & editing:** Zhihui Li, Qinlong Zheng.

## References

[1] Van der HL Pyrc K Jebbink. Identification of a new human coronavirus. Nat Med 2004;10:368–73. 15034574 10.1038/nm1024 PMC7095789

[2] Huang SH Su MC Tien N. Epidemiology of human coronavirus NL63 infection among hospitalized patients with pneumonia in Taiwan. J Microbiol Immunol Infect 2017;50:763–70. 26746130 10.1016/j.jmii.2015.10.008 PMC7105056

[3] Jin Y Zhang RF Xie ZP. Newly identified respiratory viruses associated with acute lower respiratory tract infections in children in Lanzou, China, from 2006 to 2009. Clin Microbiol Infect 2012;18:74–80. 21767329 10.1111/j.1469-0691.2011.03541.x PMC7129015

[4] van der HL Ihorst G Sure K. Burden of disease due to human coronavirus NL63 infections and periodicity of infection. J Clin Virol 2010;48:104–8. 20347384 10.1016/j.jcv.2010.02.023 PMC7108429

[5] Lambert SB Allen KM Druce JD. Community epidemiology of human metapneumovirus, human coronavirus NL63, and other respiratory viruses in healthy preschool-aged children using parent-collected specimens. Pediatrics 2007;120:e929–37. 17875651 10.1542/peds.2006-3703

[6] Moës EL Vijgen L Keyaerts E. A novel pancoronavirus RT-PCR assay: frequent detection of human coronavirus NL63 in children hospitalized with respiratory tract infections in Belgium. BMC Infect Dis 2005;5:01–10. 10.1186/1471-2334-5-6 PMC549190 15686594

[7] Bastien N Anderson KL Hart L. Human coronavirus NL63 infection in Canada. J Infect Dis 2005;191:503–6. 15655772 10.1086/426869 PMC7199484

[8] Vabret A Mourez T Dina J. Human coronavirus NL63, France. Emerg Infect Dis 2005;11:1225–9. 16102311 10.3201/eid1108.050110 PMC3320486

[9] Forster J Ihorst G Rieger CH. Prospective population-based study of viral lower respiratory tract infections in children under 3 years of age (the PRI.DE study). Eur J Pediatr 2004;163:709–16. 15372233 10.1007/s00431-004-1523-9

[10] van der HL Sure K Ihorst G. Croup is associated with the novel coronavirus NL63. PLoS Med 2005;2:e240. 16104827 10.1371/journal.pmed.0020240 PMC1188248

[11] Suzuki A Okamoto M Ohmi A. Detection of human coronavirus-NL63 in children in Japan. Pediatr Infect Dis J 2005;24:645–6. 15999010 10.1097/01.inf.0000168846.71517.ee

[12] Canducci F Debiaggi M Sampaolo M. Two-year prospective study of single infections and co-infections by respiratory syncytial virus and viruses identified recently in infants with acute respiratory disease. J Med Virol 2008;80:716–23. 18297694 10.1002/jmv.21108 PMC7167101

[13] Chiu SS Chan KH Chu KW. Human coronavirus NL63 infection and other coronavirus infections in children hospitalized with acute respiratory disease in Hong Kong, China. Clin Infect Dis 2005;40:1721–9. 15909257 10.1086/430301 PMC7107956

[14] Choi EH Lee HJ Kim SJ. The association of newly identified respiratory viruses with lower respiratory tract infections in Korean children, 2000-2005. Clin Infect Dis 2006;43:585–92. 16886150 10.1086/506350 PMC7107986

[15] Dominguez SR Robinson CC Holmes KV. Detection of four human coronaviruses in respiratory infections in children: a one-year study in Colorado. J Med Virol 2009;81:1597–604. 19626607 10.1002/jmv.21541 PMC2879166

[16] Ebihara T Endo R Ma X. Detection of human coronavirus NL63 in young children with bronchiolitis. J Med Virol 2005;75:463–5. 15648061 10.1002/jmv.20289 PMC7166887

[17] Kaiser L Regamey N Roiha H. Human coronavirus NL63 associated with lower respiratory tract symptoms in early life. Pediatr Infect Dis J 2005;24:1015–7. 16282944 10.1097/01.inf.0000183773.80217.12

[18] Smuts H Workman L Zar HJ. Role of human metapneumovirus, human coronavirus NL63 and human bocavirus in infants and young children with acute wheezing. J Med Virol 2008;80:906–12. 18360904 10.1002/jmv.21135 PMC7166566

[19] Kristoffersen AW Nordb SA Rognlien AG. Coronavirus causes lower respiratory tract infections less frequently than RSV in hospitalized Norwegian children. Pediatr Infect Dis J 2011;30:279–83. 21057374 10.1097/INF.0b013e3181fcb159

[20] Koetz A Nilsson P Lindén M. Detection of human coronavirus NL63, human metapneumovirus and respiratory syncytial virus in children with respiratory tract infections in south-west Sweden. Clin Microbiol Infect 2006;12:1089–96. 17002608 10.1111/j.1469-0691.2006.01506.x PMC7128111

[21] Wu PS Chang LY Berkhout B. Clinical manifestations of human coronavirus NL63 infection in children in Taiwan. Eur J Pediatr 2008;167:75–80. 17297612 10.1007/s00431-007-0429-8 PMC7087307

[22] Dare RK Fry AM Chittaganpitch M. Human coronavirus infections in rural Thailand: a comprehensive study using real-time reverse-transcription polymerase chain reaction assays. J Infect Dis 2007;196:1321–8. 17922396 10.1086/521308 PMC7109921

[23] Otieno GP Murunga N Agoti CN. Surveillance of endemic human coronaviruses (HCoV-NL63, OC43 and 229E) associated with childhood pneumonia in Kilifi, Kenya. Wellcome Open Res 2020;5:150. 32995556 10.12688/wellcomeopenres.16037.1 PMC7512035

[24] Cabeça TK Granato C Bellei N. Epidemiological and clinical features of human coronavirus infections among different subsets of patients. Influenza Other Respir Viruses 2013;7:1040–7. 23462106 10.1111/irv.12101 PMC4634278

[25] Hand J Rose EB Salinas A. Severe respiratory illness outbreak associated with human coronavirus NL63 in a long-term care facility. Emerg Infect Dis 2018;24:1964–6. 30226169 10.3201/eid2410.180862 PMC6154147

[26] Preiksaitis JK Brennan DC Fishman J. Canadian society of transplantation consensus workshop on cytomegalovirus management in solid organ transplantation final report. Am J Transplant 2005;5:218–27. 15643981 10.1111/j.1600-6143.2004.00692.x

[27] Fischer SA. Emerging viruses in transplantation: there is more to infection after transplant than CMV and EBV. Transplantation 2008;86:1327–39. 19033999 10.1097/TP.0b013e31818b6548

[28] Deurenberg RH Bathoorn E Chlebowicz MA. Application of next generation sequencing in clinical microbiology and infection prevention. J Biotechnol 2017;243:16–24. 28042011 10.1016/j.jbiotec.2016.12.022

[29] Mayer K Nellessen C Hahn-Ast C. Fatal outcome of human coronavirus NL63 infection despite successful viral elimination by IFN-alpha in a patient with newly diagnosed ALL. Eur J Haematol 2016;97:208–10. 26854965 10.1111/ejh.12744 PMC7163643

[30] Piñana JL Xhaard A Tridello G. Seasonal human coronavirus respiratory tract infection in recipients of allogeneic hematopoietic stem cell transplantation. J Infect Dis 2021;223:1564–75. 32860509 10.1093/infdis/jiaa553 PMC7499673

[31] Narasaraju T Yang E Samy RP. Excessive neutrophils and neutrophil extracellular traps contribute to acute lung injury of influenza pneumonitis. Am J Pathol 2011;179:199–210. 21703402 10.1016/j.ajpath.2011.03.013 PMC3123873

[32] Yan X Li F Wang X. Neutrophil to lymphocyte ratio as prognostic and predictive factor in patients with coronavirus disease 2019: a retrospective cross-sectional study. J Med Virol 2020;92:2573–81. 32458459 10.1002/jmv.26061 PMC7283791

[33] Petrilli CM Jones SA Yang J. Factors associated with hospital admission and critical illness among 5279 people with coronavirus disease 2019 in New York City: prospective cohort study. BMJ 2020;369:m1966. 32444366 10.1136/bmj.m1966 PMC7243801

[34] Erratum: Lee DW Gardner R Porter DL. Current concepts in the diagnosis and management of cytokine release syndrome. Blood 2014;124:188–95. 1533. 24876563 10.1182/blood-2014-05-552729 PMC4093680

